# Autoimmunity and Cancer: Two Stations on the Same Continuum

**DOI:** 10.7759/cureus.54317

**Published:** 2024-02-16

**Authors:** Inês Soldin, Nídia Pereira

**Affiliations:** 1 Medical Oncology, Instituto Português de Oncologia do Porto Francisco Gentil, Porto, PRT; 2 Internal Medicine, Hospital Pedro Hispano, Matosinhos, PRT

**Keywords:** immunosuppression, corticoids, biological treatments, autoimmunity, cancer

## Abstract

Introduction: Autoimmunity has been associated with different types of cancer, including hematological malignancies like lymphomas, and solid tumors. Additionally, the potential role of medication-induced immunosuppression should be considered.

Aim: Our study aimed to investigate the relationship between autoimmunity and the development of cancer, as well as the impact of immunosuppressive drugs on increasing cancer risk.

Methods: The study sample was composed of patients who developed cancer after the administration of biological agents for the treatment of autoimmune disorders. Selected patients were treated in our hospital between 1st January 2011 and 31st December 2021 and followed up in internal medicine, gastroenterology, or dermatology consult. From 434 patients with autoimmune diseases using biological agents, only 20 developed cancer, which was our final study sample. The data analysis was performed using the IQVIR package version 2.0.2 (IQVIA, Durham, NC). A p-value of <0.05 was considered statistically significant.

Results: We found a significant correlation between long-term corticosteroid therapy and an increased risk of cancer. However, the effect of biological therapies on cancer risk was not statistically significant. It's worth noting that our sample size was small, so we cannot extrapolate these findings.

Conclusions: Physicians need to be aware that treating autoimmune diseases with immunosuppressive therapies may contribute to the development of cancer. Further research is needed to determine the impact of such treatments on cancer prognosis.

## Introduction

Immune tolerance is a dynamic state that minimizes the risk of developing autoimmune diseases by making the immune system unresponsive to self-tissues [1]. The elimination of autoreactive antigens initiates in the primary lymphoid organs, such as the bone marrow and thymus, and is referred to as the central tolerance mechanism [2]. The peripheral tolerance stage eliminates autoreactive cells that manage to evade central vigilance and reach maturity. This is done through regulatory T and B cells, M2 macrophages, and tolerogenic dendritic cells [1,2]. A disruption in the balance caused by genetic predisposition, both epigenetic and genomic factors, and environmental exposure can lead to autoimmunity [2]. This can result in a permanent attack on self-cells and the release of pro-inflammatory cytokines that lead to chronic inflammation, which can promote tumorigenesis. Autoimmunity has been linked to several types of cancer, including hematological malignancies such as lymphomas and less frequently solid tumors [1-5]. Patients with systemic lupus erythematosus have a non-Hodgkin lymphoma risk increased almost three times, while those previously diagnosed with rheumatoid arthritis have a risk increased almost four times [4,6]. Patients with inflammatory bowel disease have 23 times more risk of colorectal cancer [6]. Rheumatoid arthritis, systemic lupus erythematosus, and type 1 diabetes increase the risk of multiple types of cancer [7,8]. Therefore, cancer and the autoimmune response can both be conceptualized as immune dysregulation, whose mechanisms of tolerance induction and downregulation are not completely understood [1,2,8].

Secondary immunosuppression, which is often used for the treatment of autoimmune diseases, can also be an independent risk factor for cancer development [8]. Approaches that boost the central defense stage are increasingly used to treat autoimmune diseases [8]. Inflammatory pathways are commonly targeted by the majority of agents used. Inhibitors of the tumor necrosis factor (TNF) receptor, such as adalimumab, etanercept, or infliximab, trigger downstream signaling, leading to inflammation and apoptosis [8-10]. Alternatively, immunosuppression can be due to targeting and depleting B-cells from circulation [8,11]. Rituximab is a monoclonal antibody that binds to the CD20 surface marker expressed on B lymphocytes, eliminating diseased cells from circulation [11]. Therefore, in an era of targeted immunomodulation therapies, autoimmunity, and cancer are now recognized as two stations in the same continuum of inflammation [1,8].

## Materials and methods

Subjects

We conducted a retrospective study in a hospital setting. The study's inclusion criteria were a confirmed diagnosis of an autoimmune disease that required at least one biological treatment between January 1st, 2011, and December 31st, 2021, and a diagnosis of cancer after receiving biological therapy. We included the following autoimmune diseases in our analysis: systemic lupus erythematosus, antiphospholipid syndrome, rheumatoid arthritis, psoriasis, psoriatic arthritis, vasculitis, scleroderma, serum-negative spondyloarthropathy, inflammatory bowel disease (Crohn's disease and ulcerative colitis), and Behçet's syndrome. The analysis considered all types of cancer.

We included in the analysis the biological treatments available in our hospital, which are adalimumab, etanercept, ustekinumab, infliximab, vedolizumab, and rituximab. Patients who received at least one of those agents, either in monotherapy or in combination with immunomodulators, were included in the study. Of those (N=9), three patients were under long-term corticosteroids, which was considered a minimum period of 30 consecutive days of treatment. We excluded patients who lost follow-up, who had only one of the studied diagnoses, or who developed cancer before the autoimmune disease. 

We retrieved data from the hospitalization, medical appointment, and emergency department records of Pedro Hispano Hospital in Portugal, where patients received treatment and follow-up.

Statistical analyses

The data analysis was performed using the IQVIR package version 2.0.2 (IQVIA, Durham, NC). The results were expressed as the median for quantitative data and numbers and percentages for categorical data. To compare differences in cancer prevalence for each autoimmune disease, the chi-square test was conducted and statistical significance was set at p ≤ 0.05.

A cross-tabulation analysis was performed to investigate the relationship between cancer incidence and autoimmune diseases. This analysis took into account relevant demographic and clinical factors that may act as potential confounders. These confounding factors were sex, age, smoking history, prescribed biological treatment and duration, and other immunosuppressant treatments besides biological treatment.

At the time of cancer diagnosis, the total and differential leukocyte counts were used to infer the immunosuppressant state. Reference values from our institution were used, where leukocytes were 4.0-11.0 × 10^9^/uL and neutrophils were 1.3-8.8 × 10^9^/uL.

Declaration of ethical approval

This study was performed according to the guidelines of the local ethical committee. The patient’s information was anonymized and access to the patient’s clinical notes was temporarily limited to the extent of the study. Patients undergoing clinical trials were excluded.

## Results

Patient characteristics

Between January 2011 and December 2021, our hospital treated 434 patients with autoimmune diseases using biological agents. After applying inclusion and exclusion criteria, we obtained a sample of 20 patients, of which 55% were female. The median age at the time of autoimmune disease diagnosis was 52 years (ranging from 22 to 77). The immunomodulators used in combination with biological agents are described in Table 1. Crohn's disease was the most prevalent autoimmune disease (35%). According to the Montreal Classification of Crohn's Disease, four patients with Crohn's disease were in the A2 class (17-40 years). Regarding disease location, four patients had ileal disease (L1), two had colonic involvement (L2), and one was L3 (ileocolonic). Disease behavior analysis showed that four patients had a stricturing phenotype (B2), one patient had penetrating disease (B3), and the last two had B2/B3 disease. One patient classified as A2B2 had upper gastrointestinal tract involvement and developed gastric adenocarcinoma. Two out of seven patients with Crohn's disease developed right colon adenocarcinoma, and one developed esophageal squamous cell cancer.

**Table 1:** Sample characterization. *Corticosteroid rescue therapy, used for acute disease control, was not considered here. **Breast cancer-specific risk factors: BRCA1/BRCA2 mutation status, early menarche, late menopause, high-density breast, first pregnancy after age 30, not breastfeeding, never having a full-term pregnancy.

Variables	N	(%)
Demographics		
Sex		
Female	11	55
Male	9	45
Age (years) at diagnosis of autoimmune disease		
<40 years	5	25
40-50 years	4	20
>50 years	11	55
Autoimmunity characteristics		
Autoimmune disease		
Crohn’s disease	7	35
Psoriatic arthritis	4	20
Rheumatoid arthritis	2	10
Psoriasis	2	10
Overlap of systemic lupus erythematosus + antiphospholipid syndrome	2	10
Serum-negative spondyloarthropathy	2	10
Vasculitis	1	5
Biological treatment		
Adalimumab	14	70
Etanercept	3	15
Ustekinumab	2	10
Rituximab	1	5
Treatment period (months)		
<50	13	65
50-100	3	15
>100	4	20
Immunomodulators taken at least one year before cancer diagnosis	9	45
Maintenance corticosteroid therapy*	3	25
Methotrexate	3	25
Azathioprine	3	25
Mesalazine	2	17
Sulfasalazine	1	8
Cancer characteristics		
Cancer general risk factors		
Obesity (BMI >30)	5	25
Cancer family history	2	10
Smoking status		
Never	9	45
Past	8	40
Active	3	15
Alcohol status		
Never	17	85
Past	2	10
Active	1	5
Cancer-specific risk factors		
Breast cancer hormonal and genetic risk factors**	1	5
Age (years) at diagnosis of cancer		
<40 years	1	5
40-50 years	7	35
>50 years	12	60
Primary tumor site and histology		
Luminal A breast cancer	3	15
Colon adenocarcinoma	2	10
Small cell lung cancer	1	5
Breast carcinoma ductal in situ	1	5
Luminal B breast cancer	1	5
Ovarian borderline-serous carcinoma	1	5
Esophagus squamous cell carcinoma	1	5
Gastric adenocarcinoma (intestinal-type)	1	5
Rectal adenocarcinoma	1	5
Cholangiocarcinoma	1	5
Anaplastic thyroid cancer	1	5
Malignant melanoma	1	5
Basal cell cancer	1	5
Nodular sclerosis Hodgkin lymphoma	1	5
Diffuse large B-cell non-Hodgkin lymphoma	1	5
Monoclonal gammopathy of unknown significance	1	5
Kaposi sarcoma	1	5
Stage at diagnosis		
Localized	10	50
Loco-regional	5	25
Metastatic	5	25
ECOG performance status at diagnosis		
0	8	40
1	10	50
2	2	10
Time (years) from biological treatment start date to cancer development		
<5 years	14	70
5-10 years	3	15
>10 years	3	15
Death		
Cancer-related	7	35

Adalimumab was the most prescribed biological agent (70%). For 55% of the patients, biological therapy in monotherapy was enough to achieve disease control (Table 1). Thirteen out of twenty (65%) completed treatment with biological agents for less than 50 months. Cancer diagnosis caused autoimmune disease treatment discontinuation in 65% of the patients. The median age at cancer diagnosis was 59 years (ranging from 26 to 77). About 50% of the diagnosed cancers were at an early stage, and 90% of patients had an ECOG performance status of less than 2. Breast cancer was the most frequently diagnosed neoplasia (25%), and luminal A was the most frequent type (60% of diagnosed breast cancers). Two out of five women with breast cancer were treated with adalimumab, two were under etanercept, and one underwent ustekinumab.

Five patients (25%) were obese, and 11 (55%) were ex-smokers. One patient developed esophageal cancer but was also an active smoker and an alcohol addict (120-126 alcohol units/week). No patient described occupational exposure to known carcinogens. There were also no immunosuppressed patients at the time of cancer diagnosis, considering whole blood cell count criteria. The average period between starting biological treatment and cancer was five years, with a standard deviation of 4. Seven patients died from cancer progression (Table 1). All those patients had advanced disease at the time of diagnosis.

**Figure 1:**
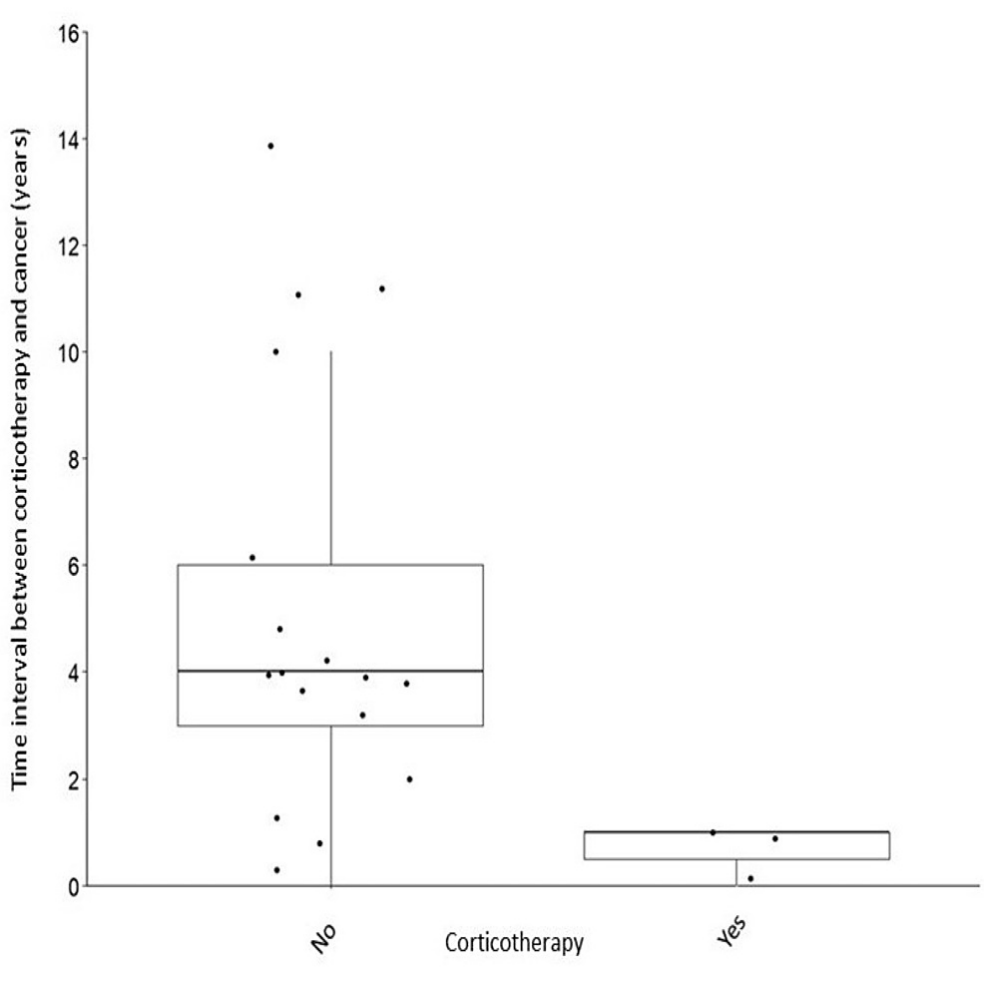
The relationship between corticosteroid therapy and cancer. Patients who had corticotherapy in the last year have an increased risk of developing cancer.

The diagnosis of cancer was statistically associated with previous chronic corticosteroid therapy (p=0.027) (Figure 1). No statistically significant association between biological treatment and posterior cancer development was found (p=0.103). The other immunomodulators used in our sample (Table 1) did not statistically correlate with cancer (p>0.05).

No statistical difference in cancer development between both sexes was found (p=0.075). In our sample, none of the considered risk factors (Table 1) was associated with cancer (p>0.05). 

Due to the limited sample size, it was not possible to assess a potential relationship between each autoimmune disease, biological agent, and any particular type of cancer. Likewise, it is not possible to conclude the critical time interval between the biological treatment start date and cancer. Although our results suggest that the prevalence of autoimmunity and cancer increases with age, the limited size of our sample did not allow us to conclude the potential relationship between the age at diagnosis of autoimmune disease and posterior cancer development. 

## Discussion

Patients with autoimmune diseases are at a greater risk of developing cancer, as reported in the meta-analysis from Pantuck et al., among other studies. Although the exact mechanism is not fully understood, it is known that chronic inflammation leads to cell injury mediated by autoantigen-specific T cells and antibodies, ultimately creating a tumorigenic microenvironment [3,12,13]. Other authors stipulate that inflammatory cells are themselves tumorigenic since tumor-associated macrophages can stimulate tumor growth and angiogenesis [8,12,14].

The first diagnosis of breast cancer is predominantly observed in women over the age of 50. However, due to a limited sample size and the prevalence of breast cancer in female patients, its connection with autoimmunity cannot be established. Therefore, the significance of underlying autoimmune diseases cannot be inferred.

In our sample, we could not prove the relationship between some well-defined autoimmune diseases and an increased risk of cancer due to the small number of patients in each condition. Although we can assume that Crhon's disease may contribute to the development of colorectal adenocarcinoma [3,5], this relationship did not show statistical significance in our study. It is crucial to investigate the effect of factors such as diet, alcohol, and smoking habits and their potential interaction with inflammatory bowel diseases in increasing cancer risk. Further studies involving a larger sample size are necessary to explore this important issue.

Lymphoma risk can be increased after treatment with adalimumab. However, it is important to consider the underlying disorder's own malignancy risk when evaluating whether the therapy increases the risk of developing cancer. Although the literature is not conclusive, a higher burden of Crhon’s disease, as well as structuring and fistulizing phenotypes, may be driving this increased risk rather than anti-TNF monotherapy. The role of long-term corticosteroid therapy in this increased risk should also be considered [15-17]. Our research has shown that the use of maintenance corticosteroids is associated with a higher incidence of cancer when compared to unexposed individuals. This is because corticosteroids decrease the number of B-cells and inhibit the immune response. They also suppress macrophage differentiation and hinder the production of pro-inflammatory molecules like prostaglandins and leukotrienes [16,17]. In contrast to the recognized role of glucocorticosteroids in determining poor clinical outcomes within established solid tumors, there are heterogeneous effects on tumor development. Therefore, there is a lack of epidemiological studies addressing the relationship between corticosteroid use and cancer risk, which probably will vary between cancer types and histological subtypes [18,19].

As well, a link between rheumatoid arthritis treated with adalimumab and solid cancers, like lung cancer, was described [20]. The extrapolation of data from rheumatoid arthritis must be done with caution, as there is a well-documented risk of developing B-cell lymphoma [15].

Although effective, treatment-induced immunosuppression can increase cancer risk in autoimmune patients [8,9]. There are proposed mechanisms of secondary tumorigenesis associated with anti-TNF agents, which include adalimumab. These mechanisms involve decreased activation of natural killer cells, promotion of apoptosis among macrophages and T cells, induction of cell cycle arrest of T cells, and inhibition of IL-1β production by monocytes [3,9,10,15]. The ceaseless release of cytokines, chemokines and free radicals, which are pro-inflammatory molecules, are also potential contributors [3,10]. One of the most studied relationships is the rituximab-secondary Kaposi sarcoma, from which we have one case [11]. It is of note that the patient tested negative for HIV. As described in the literature, our patient developed cancer immediately after starting rituximab and presented isolated skin lesions that fully resolved after rituximab discontinuation. 

As our institution is a district hospital, the study conducted had a limited sample size, which is the main drawback of this research. As the study is retrospective, there might be a concern regarding selection bias. To determine and understand the relationship between autoimmunity, treatment-related immunosuppression, and cancer development, larger and multicentric prospective studies should be conducted. It should be noted that autoimmunity and malignancy are heterogeneous, which makes it challenging to draw unifying conclusions. Larger samples will also help to verify if autoimmunity affects cancer outcomes, as suggested in the literature [4,8]. This acknowledgment will clarify if patients with previous autoimmune diseases could be candidates for immunomodulation-based cancer treatments.

## Conclusions

This study aimed to investigate the possible relationship between autoimmunity and cancer. Although both conditions frequently coexist, the exact link between them remains unclear. Therefore, doctors need to consider if autoimmunity can contribute to the development of cancer, and whether treatments for autoimmune diseases, such as biological and immunomodulatory therapies like corticosteroids, can affect cancer development or prognosis. These questions should be addressed at the time of diagnosis and when considering treatment options. Since patients with previous autoimmune diseases that subsequently develop cancer are usually excluded from new treatment randomized clinical trials, it is essential to consider primary and secondary prevention measures for neoplasms during daily clinical practice in autoimmune disease units.
